# Improvement of cellular pattern organization and clarity through centrifugal force

**DOI:** 10.1088/1748-605X/ada508

**Published:** 2025-02-13

**Authors:** Lauren E Mehanna, James D Boyd, Shelley Remus-Williams, Nicole M Racca, Dawson P Spraggins, Martha E Grady, Brad J Berron

**Affiliations:** 1 Department of Chemical and Materials Engineering, University of Kentucky, Lexington, KY, United States of America; 2 Department of Mechanical Engineering, University of Kentucky, Lexington, KY, United States of America; 3 Department of Biomedical Engineering, University of Michigan, Ann Arbor, MI, United States of America

**Keywords:** cell patterning, adhesion, centrifugation, shear flow, streptavidin, cell density

## Abstract

Rapid and strategic cell placement is necessary for high throughput tissue fabrication. Current adhesive cell patterning systems rely on fluidic shear flow to remove cells outside of the patterned regions, but limitations in washing complexity and uniformity prevent adhesive patterns from being widely applied. Centrifugation is commonly used to study the adhesive strength of cells to various substrates; however, the approach has not been applied to selective cell adhesion systems to create highly organized cell patterns. This study shows centrifugation as a promising method to wash cellular patterns after selective binding of cells to the surface has taken place. After patterning H9C2 cells using biotin-streptavidin as a model adhesive patterning system and washing with centrifugation, there is a significant number of cells removed outside of the patterned areas of the substrate compared to the initial seeding, while there is not a significant number removed from the desired patterned areas. This method is effective in patterning multiple size and linear structures from line widths of 50–200 μm without compromising immediate cell viability below 80%. We also test this procedure on a variety of tube-forming cell lines (MPCs, HUVECs) on various tissue-like surface materials (collagen 1 and Matrigel) with no significant differences in their respective tube formation metrics when the cells were seeded directly on their unconjugated surface versus patterned and washed through centrifugation. This result demonstrates that our patterning and centrifugation system can be adapted to a variety of cell types and substrates to create patterns tailored to many biological applications.

## 1. Introduction

Cell patterning is a common technique used to organize cells into complex structures (*e.g.* lines, shapes, *etc*) found in existing tissue [1, 2]. Most *in vitro* engineered tissues involve organizing cells on a substrate comprised of one or more extracellular matrix components (ECM) found in tissue [3, 4]. While there are currently many approaches to cell patterning on 2D substrates, most focus on two approaches: topographical cues on the substrate (lithography [5–8]) or environmental cues (optical [9–11], magnetic [12, 13], electrical [14, 15], or acoustic [16–18] stimulation) to guide cells into a desired pattern organization. These methods can often be expensive and have lengthy fabrication processes prior to cell seeding [19, 20]. Additional strategies combining microfluidics with patterned microwells have been developed to capture cells in desired microarrays [21, 22]. The major drawback with this approach is that cells become immobilized to the microwells, limiting their long-term behavior. Specific cellular assays, such as cancer cell migration and invasion assays, require patterning techniques that do not confine cells to a specific location or limit their natural dynamic processes. Confinement-free cell patterning has successfully been achieved using microfluidic techniques, such as through fluidic cell trapping and with tape-assisted micromolds that guide the location of cell adhesion [23, 24]. While these methods can achieve high resolution patterns, some have difficult sample handling and can be limited by the geometries which can be produced.

Additional approaches have been studied to encourage cells to bind to selective areas of a substrate and create patterns based on their adhesion strength [25]. In this type of system, a cell adheres strongly to one location of the substrate, the patterned region, while having a much weaker adhesion in another location, the unpatterned region. This strategy of patterning based on adhesion strength is common using various proteins or antibody-antigen interactions, where molecules with a high binding affinity for one another are added to the cell or substrate surface to promote adhesion [26–28]. Additional adhesion-based patterning methods incorporate changes to the hydrophobicity of various parts of the substrate, encouraging or discouraging cell adhesion in specific regions [29, 30]. DNA cell patterning techniques also utilize selective binding, involving hybridization of synthetic DNA strands in the cell membrane that have a high binding specificity to complementary DNA strands on the substrate surface. By tailoring the location of these complementary DNA strands on the substrate, patterned features ranging in size from 10–1000 μm wide can be achieved [31, 32]. Current adhesive cell patterning methods have achieved numerous geometries (lines, dots, organ features, etc.) that are tailorable for biological outcomes [25, 31, 33]. In this study, we chose to investigate straight-line pattern structures, as they are simplistic in modeling patterning and cell removal while providing relevancy to tube forming cell lines.

Adhesive cell patterning provides potential for designing highly organized 2D cellular layers for scaleup in tissue construct formation. In this study, we use the binding motif of biotin and streptavidin, known for forming one of the strongest non-covalent biological interactions, as a model for adhesive cell patterning [34, 35]. The location of the biotin-streptavidin binding motif on the substrate controls how cells will strongly or weakly adhere based on where the bioactive molecules are present. Biotin-streptavidin has been demonstrated as a cell patterning approach with functional outcomes, motivating its use as the model of this study [25, 33, 36]. The necessary functional outcome, such as cell alignment, migration behavior, or tube formation, is an important parameter when designing cell patterning systems for tissue construct designs. For example, primary myogenic progenitor cells (MPCs) have been patterned onto collagen I, and rapidly aligned into functional parallel myotubes, to aid in large volume muscle repair [33]. Adhesive patterning systems differ from microcontact printed systems, which rely on printing extracellular matrix materials onto a non-adhesive culture surface or printing permanent non-adhesive patterns to create topographical ques on a non-adhesive culture surface. Instead, pattering with bioactive molecules, like biotin-streptavidin, creates a temporary adhesive guide that aids in initial cell placement but does not confine cells long-term from migrating and behaving naturally [33].

**Figure 1.**
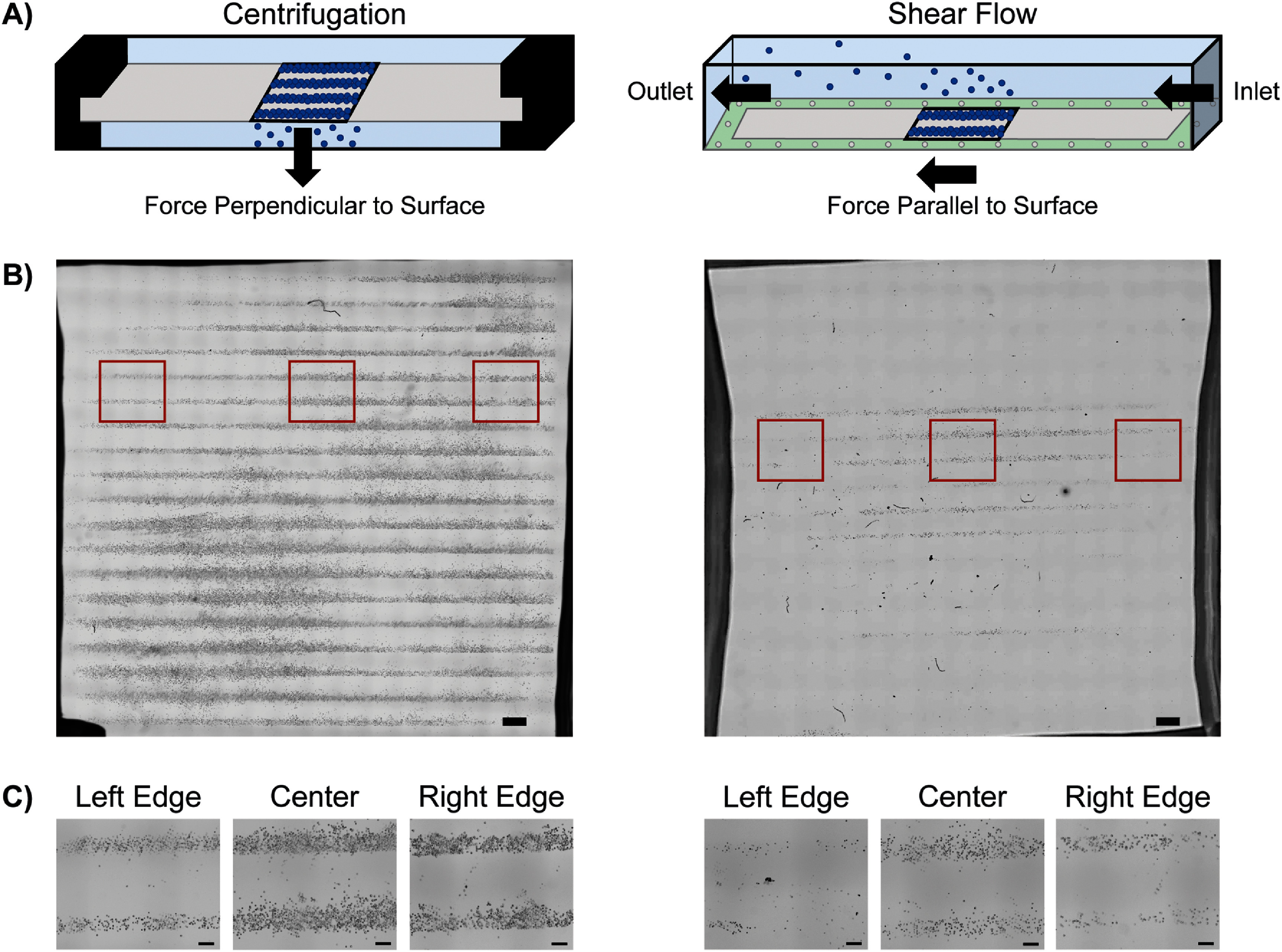
Centrifugation Versus Shear Flow for Washing Cellular Patterns. (A) Schematic representation of the centrifugation (left) and shear flow (right) washing systems for cell patterns. (B) Full well images of H9C2 cells patterned in 200 μm line widths and washed through the centrifugation (left) and shear flow (right) washing systems using brightfield microscopy and 4x magnification. The centrifugation washing system used 43.3 RCF in 1 min increments. The shear flow washing system was applied using a parallel plate flow chamber at 50 ml min^−1^ (69.75 dynes cm^−2^) for 15 s. (C) Representative images of cell patterns on the left, center, and right edges of the full well in (B). Image locations are denoted by red boxes. Scale bars represent 1000 μm in (B) and 200 μm in (C).

In all adhesion-based patterning systems (biotin-streptavidin, antibody-antigen, DNA patterning, hydrophobic-hydrophilic), it is crucial to remove the weakly adhered cells outside of the patterned areas while retaining the cells bound within the patterns to achieve specific long-term functional outcomes. The most common washing systems used to differentiate cell adhesion strength involve shear flow, in which fluid flow initiates a shear stress on the cell parallel to the surface [37, 38]. When this shear-induced delamination force is stronger than the adhesive strength of the cell to the surface, the cell is removed and washed away. Shear flow can be initiated in a variety of ways through pipettes, pumps, and microfluidic flow devices [38]. While shear flow systems can be effective for cell removal, they often involve non-uniform shear forces, complex set-ups, and protocol ambiguity that leads to challenges with reproducibility. We have developed a washing approach using centrifugation that improves consistency in cell removal based on adhesive binding of biotin-streptavidin and cell adhesion strength compared to shear flow (figure 1(A)).

Centrifugation has long been used in cell culture protocols to collect trypsinized cells in suspension between passages, which provides a baseline for using centrifugation for cell adhesion systems [39, 40]. While centrifugation has been applied in cell passaging procedures for cells in suspension, thoughtful design is required in using it for cell adhesion systems where balancing the force of adhesion and force of centrifugation plays a key role in maintaining cell health and function. Centrifugation has been used in biomaterials research to determine the adhesive strength of a cell to a particular substrate by applying a perpendicular force to the surface [41, 42]. However, these methods have not been widely applied to cell patterning systems, specifically those which rely on cell adhesion to create patterned structures. We expect centrifugation to be advantageous over shear flow due to its uniform detachment force across the substrate, straightforward setup, precise temperature control, procedure reproducibility, and setting tunability based on the variations in cell adhesion strength to the substrate. Cell removal through centrifugation allows for tailored cell patterned structures to be formed without impacting cell viability and function, making it robust for use in a variety of biological applications. This study focuses on the comparison of centrifugation and shear flow washing methods in conjunction using biotin-streptavidin patterning as a model adhesive patterning system for forming cell structures.

## 2. Materials and methods

### 2.1. Cell culture

Rat cardiomyoblasts (H9C2) (ATCC, Manassas, VA), human primary myogenic progenitor cells (MPC) (NDRI), and human umbilical vein endothelial cells (HUVEC) (ATCC, Manassas, VA) were cultured at 37˚ C and 5% CO_2_. H9C2 cells were cultured in Dulbecco’s Modified Eagles Medium (DMEM) (Millipore Sigma, Burlington, MA) supplemented with 10% fetal bovine serum (FBS) (Corning, Corning NY), 1% penicillin-streptomycin (VWR, Radnor, PA), and 1% antibiotic-antimycotic (Thermo Fisher, Waltham, MA). HUVECs were cultured in EGM^TM −2^ endothelial growth media BulletKit^TM^ (Lonza, Basel, Switzerland) supplemented with 1% penicillin-streptomycin and 1% antibiotic-antimyocotic. Both cell lines were cultured in 75 cm^2^ culture flasks (VWR, Radnor, PA) to approximately 90% confluency and passaged using 0.25% Trypsin-EDTA (Thermo Fisher, Waltham, MA). MPCs were obtained from the National Disease Research Exchange (Res Code DPEC6 001) and cultured in Hams F-12 Nutrient Mixture (Thermo Fisher, Waltham, MA) supplemented with 20% FBS (Biotechne, Minneapolis, MN), 1% penicillin-streptomycin, and 1% antibiotic-antimycotic. A stock solution of 100 μg basic fibroblast growth factor (bFGF) (Peprotech, Rocky Hill, NJ) was dissolved with 5 mg bovine serum albumin (BSA) (Millipore Sigma, Burlington, MA) and 1 ml phosphate buffered saline (PBS) (Millipore Sigma, Burlington, MA). The growth media was supplemented with 1 μl of the bFGF stock solution per 10 ml media. MPCs were cultured in 100 × 20 mm Primaria culture flasks (Corning, Corning NY) to 20%–50% confluency and passaged using 10x Trypsin-EDTA (Thermo Fisher, Waltham, MA) diluted to 1x concentration in PBS.

During patterning experiments, H9C2 cells were seeded on epoxy coated glass slides (CEL Associates, Pearland, TX). HUVECs were seeded on Matrigel growth factor reduced, phenol red-free, and LDEV-free (Corning, Corning NY) coated on epoxy glass slides. Matrigel aliquots were thawed on ice overnight at 4 °C, then added to 4 cm^2^ chip-clip wells to solidify at 37 °C for 30 min. MPCs were seeded on Biocoat collagen 1 coated 4-well slides (Corning, Corning NY). All solutions throughout the patterning process were prepared in 1x PBS, except for when using MPCs in which solutions were prepared in 1x PBS supplemented with 1% glucose and 1% penicillin-streptomycin.

### 2.2. Substrate patterning with biotin-streptavidin

The substrate of interest was patterned as previously described [25, 33]. The substrate was coated with 5 mg ml^−1^ BSA for 1 h, then washed with Deionized (DI) water and air dried. A 100 mg ml^−1^ TFPA-PEG3-Biotin (Thermo Fisher, Waltham, MA) in DMSO (VWR, Radnor, PA) stock solution was prepared, then diluted with PBS to create a 1 mg ml^−1^ working solution. A 20 μl volume of the working solution was added to the patterned region on the given slide, then covered with a chrome on quartz photomask etched with the desired pattern size (University of Louisville, KY) and exposed to a 365 nm UV light (Thor Labs, Newton, NJ) with 1 mW cm^−2^ intensity for 5 min. The sample and photomask were each washed with DI water and air dried. A solution of 20 μl ml^−1^ Streptavidin-Cy3 (Invitrogen, Waltham, MA) was added to the well for 30 min and the sample was rinsed again with DI water and air dried. Biotin-streptavidin patterns on the substrate surface were verified using fluorescent microscopy on an Eclipse Ti-U inverted microscope (Nikon, Minato City, Tokyo, Japan) and the fluorophore concentration was analyzed using a microarray scanner (Affymetrix 428, Santa Clara, CA). Pattern sizes studied were 200, 100, and 50 μm line widths with 800 μm spacing between lines. Patterned slides scanned with the array scanner were analyzed with ImageJ to determine the pixel intensity within the patterned and unpatterned areas of the slide. In the middle of the sample, 10 data points were collected within 4 patterned and 4 unpatterned lines and the average pixel intensity of the data points for each region were then used to calculate the Cy3 fluorophore concentration using a Cy3 calibration slide (Full Moon Biosciences, Sunnyvale, CA).

### 2.3. Cell surface modification with biotin

Each cell line was trypsinized and washed twice for 3 min at 400 RCF with PBS (Eppendorf 5702R, Hamburg, Germany). H9C2s were washed at 4 °C and kept on ice while HUVECs and MPCs were washed and biotinylated at room temperature to maintain maximal viability. Biotinylation of the cell surface was performed by adding 1 mM Sulfo-NHS-LC-Biotin (Thermo Fisher, Waltham, MA) to the cell pellet at 250 μl per 1 million cells. The cells were incubated for 40 min, then washed twice with PBS for 3 min at 400 RCF. A 1:2000 Hoechst 33343 nuclear stain (Thermo Fisher, Waltham, MA) was added to the pellet and the cells were incubated for an additional 20 min, followed by two washes with PBS. The cell pellet was resuspended in PBS and the cells were seeded on the patterned well on the slide for 40 min in darkness by covering with aluminum foil. Cell seeding density on the slide was determined based on the cell diameter and the patterned well size to maximize the number of bound cells to the patterns. H9C2s were added to a 4 cm^2^ epoxy-BSA well at a seeding density of 1.25 × 10^5^ cells cm^−2^. MPCs were added to a 1.7 cm^2^ collagen I well at a seeding density of 1.18 × 10^5^ cells cm^−2^. HUVECs were added to a 4 cm^2^ Matrigel well at a seeding density of 4.69 × 10^4^ cells cm^−2^.

### 2.4. Centrifugation for pattern washing

Cell patterned slides were washed using centrifugal force in a centrifuge with two 96-well plate attachments (Jouan CR 412, Saint-Herblain, France) to remove cells weakly adhered in unpatterned regions while retaining cells strongly adhered in patterned regions. The centrifuge basin as well as all attachments were sterilized with 70% ethanol before and after use to minimize contamination risk. Slides were washed in a sterile 4-well rectangular dish (Nunc, Thermo Fisher, Waltham, MA) that fit in the 96-well plate attachments, with one slide rinsed per well of the dish. The slides were inverted cell-side down, placed in 3D printed pieces pre-sterilized in 70% ethanol, fully submerged in 1x PBS, and centrifuged at 43.3 RCF for 1 min (SI 1). Once washed, the slides were removed from the rectangular dish and placed in a new sterile dish, cell-side up, with 5 ml fresh PBS so that cell patterns could be easily imaged. The entire 4 cm^2^ patterned area of the slide was also imaged using the large scan tiling feature on the Eclipse Ti-E (Nikon, Minato City, Tokyo, Japan) inverted microscope.

### 2.5. Determination of cell detachment force during centrifugation

The force of cell detachment from the biotin-streptavidin patterned surface is proportional to the force of centrifugation applied to the system. This force of centrifugation is optimized to introduce a detachment force great enough to remove weakly bound cells in the unpatterned regions, while not great enough to remove strongly adhered cells in the patterned regions. The cell detachment force under centrifugation was determined using the equation:

\begin{equation*}{F_D} = \left( {{\rho _{cell}} - {\rho _{medium}}} \right)*{V_{cell}}*{F_c}\end{equation*}

where *F_D_* is the detachment force experienced by the cell (pN), *ρ_cell_* is the mass density of the cell (g cm^−3^), *ρ_medium_* is the mass density of the washing media (g cm^−3^), *V_cell_* is the volume of the cell (cm^3^), and *F_c_* is the relative centrifugal force (RCF, m s^−2^) [43]. The detachment force scales linearly with the relative centrifugal force.

A Stock Isotonic Percoll® (SIP) solution (Cytiva, Marlborough,MA) with 5 types of beads of different known mass density from a Density Marker Bead (DMB) kit (Cospheric, LLC, Santa Barbara, CA; DMB-kit) was centrifuged to separate the beads into distinct bands within a 1 ml tube. A calibration curve was created relating the known densities to each band height from the bottom of the tube (supplementary figure 5). The H9C2 cell mass density was calculated from the calibration curve by relating the cell band height in the Percoll solution found experimentally. Additional details on this process are found in SI 3.

### 2.6. Shear flow for pattern washing

The shear flow washing system was created using a parallel plate flow chamber with a 1.0 cm flow path width and 0.01 in gasket thickness (Glycotech #31-010, Gaithersburg, MD). The flow chamber was sealed to the patterned slide using a vacuum pump (Gast, Benton Harbor, MI). A syringe pump with two 60 ml syringes (New Era, NE-4000, Farmingdale, NY) was used to control the volumetric flow rate and washing time of PBS across the patterned slide. The flow chamber device was transparent, allowing the patterned slide to be viewed and imaged as it was washed with PBS. TinyTake videoing software was used to capture the removal of cells from the slide in real-time.

### 2.7. Analysis of cell patterns after washing

All image analysis was completed using ImageJ (NIH). All patterning studies were completed with n = 3 independent replicates. Graphical figures were plotted as the mean with error bars representing the standard deviation across the replicates. ImageJ ‘Threshold’ and ‘Analyze Particles’ features were used to count the number of cells in the entire image as well as within the patterned and unpatterned areas of each image before and after washing with centrifugation and shear flow, which was used to calculate the cells cm^−2^ retained in each region. This cell counting method was verified to be within 5% error of the manual cell count. The cell count of each sample represented an average of 5 images per slide. The average of each sample was then used for the n = 3 independent sample mean and standard deviation.

### 2.8. Cell viability and function assays

H9C2, MPC, and HUVEC cells were stained using calcein AM (Invitrogen, Waltham, MA) and ethidium homodimer-1 (Eth-1) (Invitrogen, Waltham, MA) as a live/dead assay to determine if patterning and centrifugation impacted cell viability (SI 7). MPCs and HUVECs, cell lines that naturally form tube structures, were stained using their respective staining protocols as a long-term functional assay to ensure that patterning and centrifugation did not alter their ability to form tubes (SI 8).

### 2.9. Statistical methods

A paired Student’s t-test was used to compare the adhesion of cells remaining in the patterned and unpatterned areas of the sample before and after centrifugation or shear flow washing. An independent Student’s t-test was used to compare the adhesion of cells remaining in the patterned and unpatterned areas of a sample between various speed, time, and line width conditions. An independent Student’s t-test was also used to compare results for patterning + centrifugation controls to negative controls in all viability assays. For all t-tests, significance was reported as * p < 0.05, ** p < 0.01, and *** p < 0.001.

## 3. Results and discussion

Patterned adhesive surfaces are only effective in creating organized cellular structures when paired with an effective strategy for the removal of cells from undesired regions of the surface. This study establishes a washing method using centrifugation to create more uniform, large scale tissue structures for applications in cellular arrays [44–46], cardiovascular tissue patches [47, 48], and two-dimensional precursors to skeletal muscle constructs [33, 49–51]. Centrifugation strategies which utilize commonplace benchtop tools improve on organized cell structures formed from the shear flow washing systems, a more commonly used approach in the literature (figure 1(A)) [38, 52, 53]. The combination of an adhesive patterning method with a reliable and reproducible washing method is critical for widespread application in regenerative tissue fabrication.

### 3.1. Centrifugation outperforms shear flow in selective cell removal and pattern uniformity

The key difference between centrifugation and shear flow as a washing method of cell patterns is the direction of force applied to the cell which removes it from the slide (figure 1(A)). Because the modality of force in each system is different, cells at the material interface will fail at different levels under shear (parallel) versus normal (perpendicular) loading. When the slide is inverted in a 4-well dish and centrifuged at a constant speed, the force applied to the cells on the slide is perpendicular to and away from the slide. This perpendicular detachment force is advantageous for cell removal in that it applies a uniform force on each cell across the slide and the cells do not interact greatly with neighboring cells when being removed. Conversely, shear flow washing using a parallel plate flow chamber device creates a lateral force from one side of the slide to the other, resulting in a less uniform force acting on each individual cell. To confirm appropriate fluid flow, a shear flow velocity profile was developed experimentally, that matched the expected model for a Newtownian fluid in the laminar flow regime (SI 5, supplementary figure 7). Based on our experimental data, less force is required for cell removal when the detaching force is acting perpendicular rather than parallel to the cell adherent plane. Due to the fundamental differences in the force of cell detachment, it is more meaningful to compare the outcome of cell adhesion from each washing approach. Therefore, the percentage of cells that were retained after washing using centrifugation versus shear flow relative to their initial seeding density gives a more direct comparable metric between washing methods and is a clear indication of patterning success.

During shear flow cells are predominantly removed using a rolling mechanism, where a propulsive force and torque are exhibited on the cell by the flowing fluid [54–56]. When these forces applied to the cell through fluid flow exceed the adhesive forces of the cell bound to the slide, the cell is removed and washed away. This rolling action can have more variability for removal depending on variations in cell adhesion strength. Furthermore, removal through shear flow can decrease cell retention, as the cell delaminating from the surface is more likely to physically interact with neighboring cells, causing their removal as well. This process introduces additional factors influencing cell removal rather than from the forces of fluid flow alone.

The uniformity of the cell patterns achieved are not only a direct result of the uniformity of the force applied to the cells on the slide, but also the set-up of each washing approach, as seen in the large scan tiled images of the entire patterned slide (figure 1(B)). Centrifugation procedures do not negatively impact cell adhesion as seen in the set-up and removal procedures for the shear flow approach. Centrifugation results in more consistent patterns across the entire patterned surface, especially near the edges, and allows for the samples to be re-cultured after the desired cell patterns are achieved (figure 1(C)). The number and size of cell patterns achieved through centrifugation are only limited by the size of the slide being patterned and the dish it fits into, which could be tailored to larger patterned surfaces with only slight modifications. Alternatively, shear flow pattern sizes are greatly limited by the size of the flow chamber device, which also adds challenges in applying and removing the gasket with a vacuum seal before and after use. Shear flow considerably affects the pattern clarity around the edges of the device and makes it much more difficult to retain an adequate number of cells to re-culture (figure 1(C)).

Shear flow was tested both parallel and perpendicular to the direction of the surface patterns at an optimal flow rate of 50 ml min^−1^ (69.75 dynes cm^−2^) for 15 s. The optimal flow rate was chosen to maximize unpatterned cell loss above 90% while maintaining over 50% of patterned cells (supplementary figure 6(A)). Flowrates above 50 ml min^−1^ were not explored, as they were less effective in retaining patterned cells and studies have shown that cell viability decreases with increasing shear stress [57–59]. There is literature to support that increasing shear stress in extrusion-based 3D bioprinting decreases cell viability as well [60–62]. When cells were exposed to 50 ml min^−1^ shear flow, there was an immediate detachment response within 15 s, with little change in cell detachment for either the patterned or unpatterned condition after this timepoint (supplementary figure 6(B)). Therefore, 15 s was chosen as the optimal exposure time for shear flow studies.

**Figure 2.**
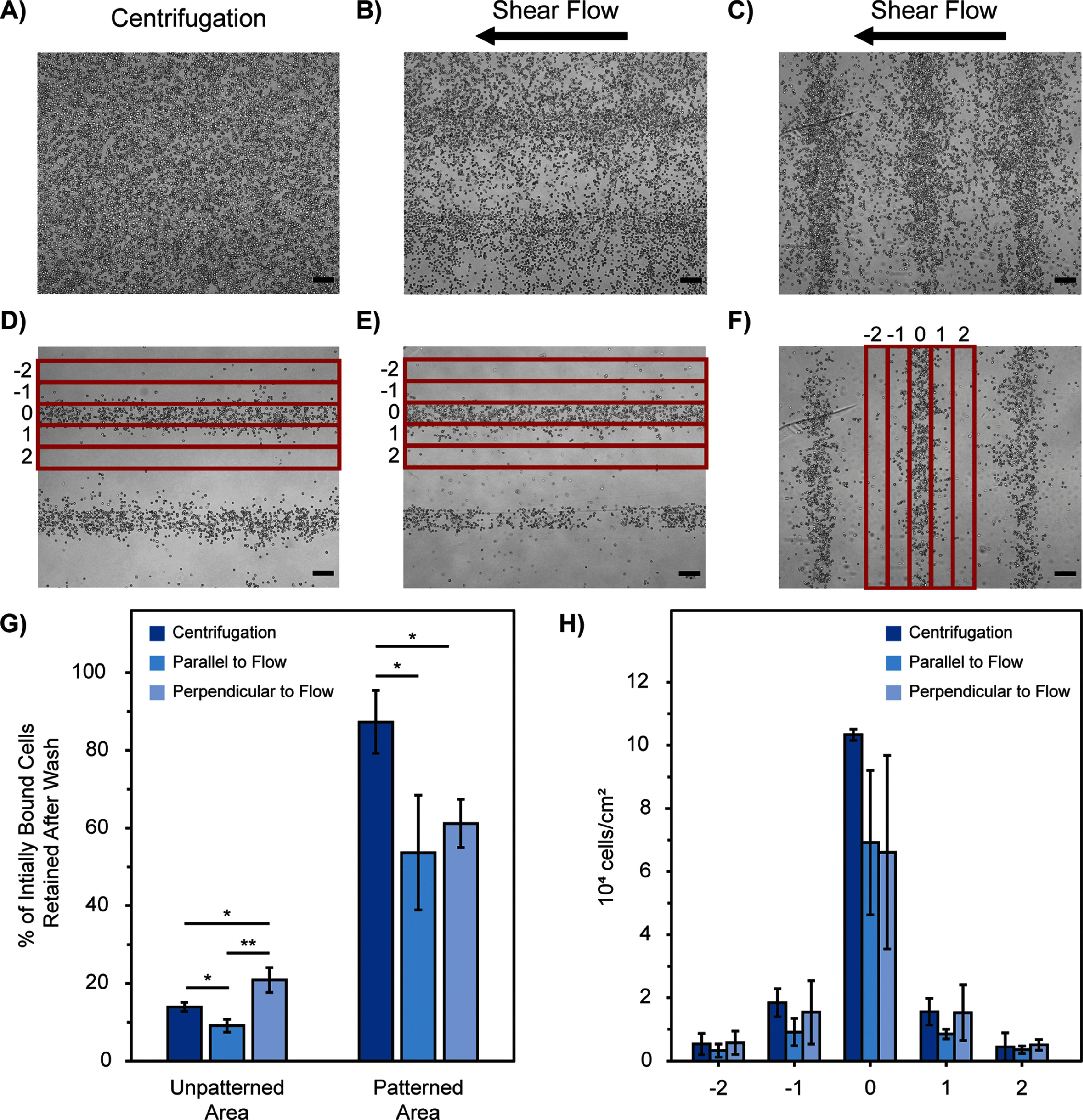
Directionality of Cell Patterns to Shear Flow Impacts Pattern Clarity. (A) Representative images of H9C2 cells patterned in 200 μm line widths before and (D) after washing using centrifugation at 43.3 RCF in 1 min increments. Cell patterns were visualized using brightfield microscopy and 4x magnification. (B) Representative images of H9C2 cells patterned in 200 μm line widths parallel to the direction of shear flow before and (E) after washing at 50 ml min^−1^ (69.75 dynes cm^−2^) for 15 s. Cell patterns were visualized using brightfield microscopy and 4x magnification prior to removal of the flow chamber device. (C) Representative images of H9C2 cells patterned in 200 μm line widths perpendicular to the direction of shear flow before and (F) after washing at 50 ml min^−1^ (69.75 dynes cm^−2^) for 15 s. Cell patterns were visualized using brightfield microscopy and 4x magnification prior to removal of the flow chamber device. (G) Percentage of initially bound cells that remain adhered in the patterned and unpatterned regions of the sample after the washing conditions described in (A)-(F). Data is shown as mean ± standard deviation of n = 3 independent samples for each condition. Significance testing compared both shear flow conditions and centrifugation for the cells retained in and out of the patterned regions using a two-group independent t-test (* p < 0.05). (H) Cell adherance in 5 binned regions surrounding a single cell pattern for each of the washing conditions described in (A)-(F) (marked in red on bottom images). Data is shown as mean ± standard deviation of n = 3 independent samples for each condition. Significance testing compared both shear flow conditions and centrifugation for each bin using a two-group independent t-test (* p < 0.05, ** p < 0.01, *** p < 0.001). The absence of * is considered a nonsignificant p-value comparison. All scale bars represent 200 μm.

In both shear flow directions, shear flow retained over 26% less of the initially bound cells within the patterned area compared to samples washed with centrifugation (figure 2(G)).

To determine if shear flow directionality was an important parameter for the pattern washing system, each set-up was studied to see if pattern clarity would be altered depending on if flow was parallel or perpendicular to the surface patterns (figures 2(E) and (F)). There was not a significant difference in the number of cells retained in patterned areas after shear flow washing in either orientation; however, there was more variability in the cell adhesion for patterns perpendicular to the direction of flow, especially in the bin of the desired cell pattern (Bin 0) and those directly surrounding it (Bins −1 and 1) (figures 2(D)–(F) and (H)). Centrifugation washing was able to achieve greater cell retention in the patterned areas of the slide (Bin 0) with less variability compared to both shear flow approaches and without increasing the cell retention in the surrounding bins (Bins −1 and 1). While there may be regions with sharper cell patterns using parallel shear flow, there are also regions with lower cell density or absence of cells. Pattern inconsistency and variability of cell density (Bin 0) (figure 2(H)) was greater using both shear flow methods regardless of the pattern direction. Therefore, shear flow washing was shown to be less reliable in producing crisp cell patterns across many replicates. Centrifugation creates clear cell patterns with high cell density. There were significantly more cells retained within the patterns (figure 2(G)) with less variability (Bin 0) (figure 2(H)), making it more reliable as a pattern washing approach for straight line structures. This comparison between shear flow and centrifugation is expected to be similar in more complex pattern geometries. Shear flow creates variability in the force acting on each individual cell, impacting overall cell pattern density and consistency. A washing approach with more uniformity in force, such as with centrifugation. would be favorable to achieve consistent pattern fidelity, especially with smaller and more detailed features.

Cell retention directly corresponds to the method of cell removal; cell removal during centrifugation is solely dependent on the perpendicular detachment force, whereas shear flow creates a parallel force that causes cells to collide with neighboring cells during delamination. Most cell patterning applications require a long-term functional outcome. Some cell lines require extreme patterning precision compared to others (e.g. neuronal networks, skeletal muscle fibers, corneal repair) to achieve this functional result [18, 63–65]. For these situations where high cell surface density is necessary, centrifugation washing is recommended, as this method allows for more precise cell removal.

The direction of shear flow washing does not significantly alter or improve the pattern clarity, although there is an increase in variability of patterns washed perpendicular to flow (figure 2(H)). When cells are removed in the unpatterned areas with flow parallel to the patterns, they roll into other cells with a similar weak adhesion strength to the surface, causing their delamination as well. However, when cells are subjected to a shear flow perpendicular to the patterns, they are more likely to roll into cells in the patterned areas that are more strongly adhered to the surface, therefore creating a barrier for them to continue to flow and become detached. This barrier results in significantly greater cell retention in the unpatterned areas when washed with shear flow perpendicular compared to parallel to the patterns (figure 2(G)).

### 3.2. Cell mass density is a key factor in calculating cell detachment force during centrifugation

Centrifugation has been shown previously as a method for detaching cells from a surface in a controlled and incremental manner [41–43]. Unfortunately, the detachment force is highly sensitive to several quantities in addition to the relative centrifugal force, specifically the mass density of the media, cell type, and the volume of each cell. Because of the small size scale and similar densities among cells and media, a slight variation will result in drastically varying force values. Measuring the mass density of the selected cell type improves the accuracy of determining cell detachment force under centrifugation. A Percoll® density gradient formed under centrifugation provides a simple, repeatable method of measuring these cell mass densities (supplementary figure 4). From equation (1) (Materials and Methods 2.5) and the experimentally determined cell mass density (SI 3), the mean cell detachment forces were calculated to be 49.4, 198, and 445 pN at centrifugal speeds of 43.3, 173, and 390 RCF, respectively. The variable values used in this equation were *ρ*_cell_ = 1.029 g cm^−3^, as calculated in this section and SI 3; *ρ_medium_*= 1.005 g cm^−3^ for the density of 1x PBS, experimentally determined through mass measurements and confirmed with reported literature values [66, 67]; *V_cell_*= 4847 μm^3^ for the volume of the H9C2 cells, calculated assuming a spherical shape with an average diameter of 21 μm; and *F_c_* = 43.3, 173, and 390 RCF.

The results obtained in this study for the mass density of the H9C2 cells are consistent with what is expected and similar to the density of the washing media used (PBS). The detachment force in pN is comparable to other cellular centrifugation assays in literature [41, 68, 69]. Additionally, units of normal force are not as universally comparable as typical mechanical detachment techniques, typically obtaining values of stress [70–72]. The technique still allows for successful detachment of localized cell adhesion while preserving cell viability of remaining cells and does so without generating any shearing effects on existing cells.

### 3.3. Minimizing centrifugation speed and duration maximizes viable cell adhesion

**Figure 3.**
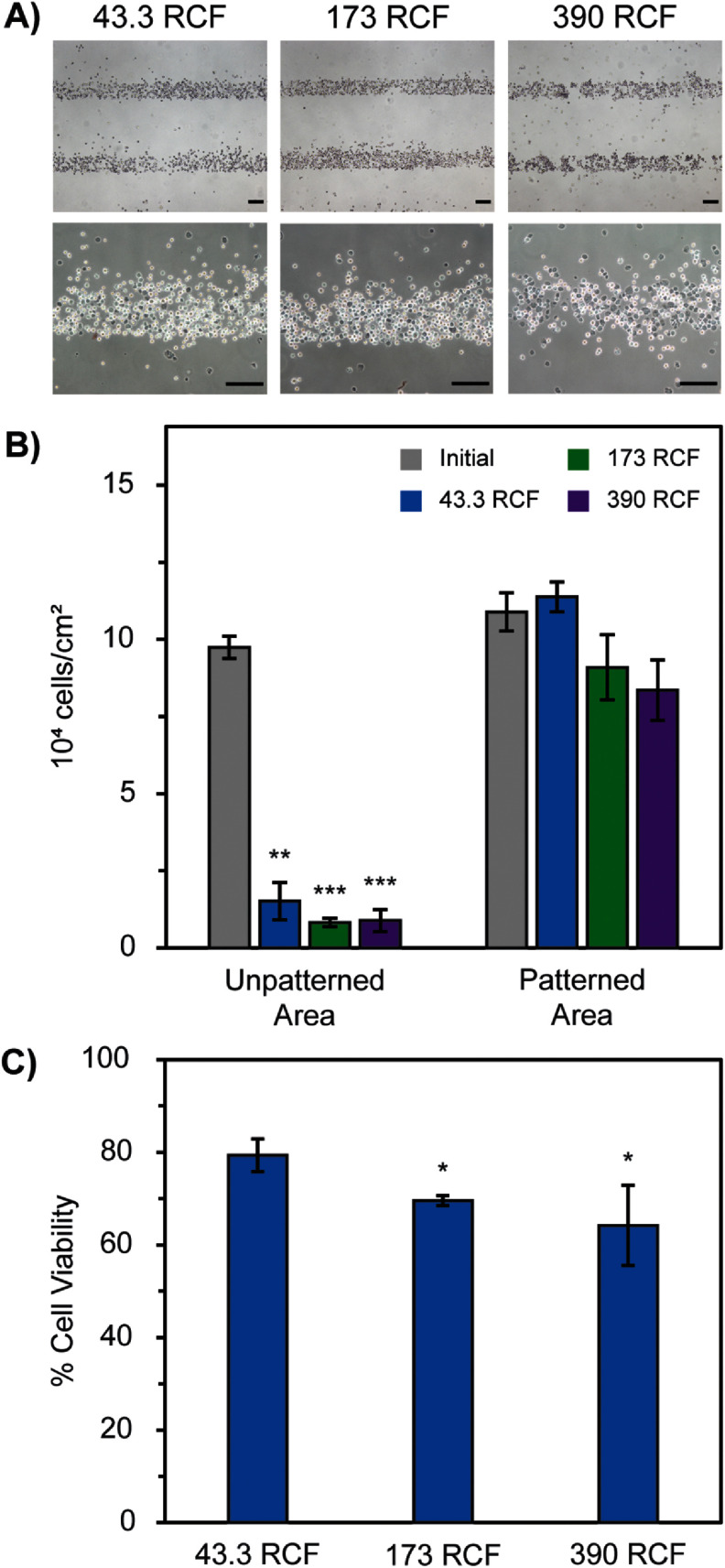
Centrifugation Speed Affects Cell Adhesion and Viability of H9C2 Cells. (A) Representative images of H9C2 cells patterned in 200 μm line width patterns and washed with centrifugation for 5 min at 43.3, 173, and 390 RCF, which correspond to cell detachment forces of 49.4, 198, and 445 pN respectively, using brightfield microscopy and 4x magnification (top row). Samples were stained with Trypan Blue to determine the percentage of cells that had damage to their membranes (bottom row). (B) Cell adherence within the patterned and unpatterned areas of each image before and after centrifugation washing of the conditions described in (A). Data is shown as mean ± standard deviation of n = 3 independent samples for each speed tested. Significance testing compared the initial cell adherence in each region before and after centrifugation at each speed using a paired two-group t-test (* p < 0.05, ** p < 0.01, *** p < 0.001). No * above data is considered nonsignificant. (C) Cell viability of patterns determined from Trypan Blue staining after centrifugation washing of the conditions described in (A). Data is shown as mean ± standard deviation of n = 3 independent samples for each speed tested. Significance testing compared the cell viability after centrifugation at 173 RCF and 390 RCF to the lowest speed of 43.3 RCF using an independent two-group t-test (* p < 0.05, ** p < 0.01, *** p < 0.001). The absence of * is considered a nonsignificant p-value comparison. All scale bars represent 200 μm.

In order to use centrifugation as a reliable cell patterning method, an optimized, consistent system must first be established for reproducibility. Studies for the optimal centrifugation speed showed that there was a significant difference in the percentage of cells retained in the unpatterned areas before and after washing when centrifugation speeds were varied while holding the centrifugation time constant at 5 min (figures 3(A) and (B)).

**Figure 4.**
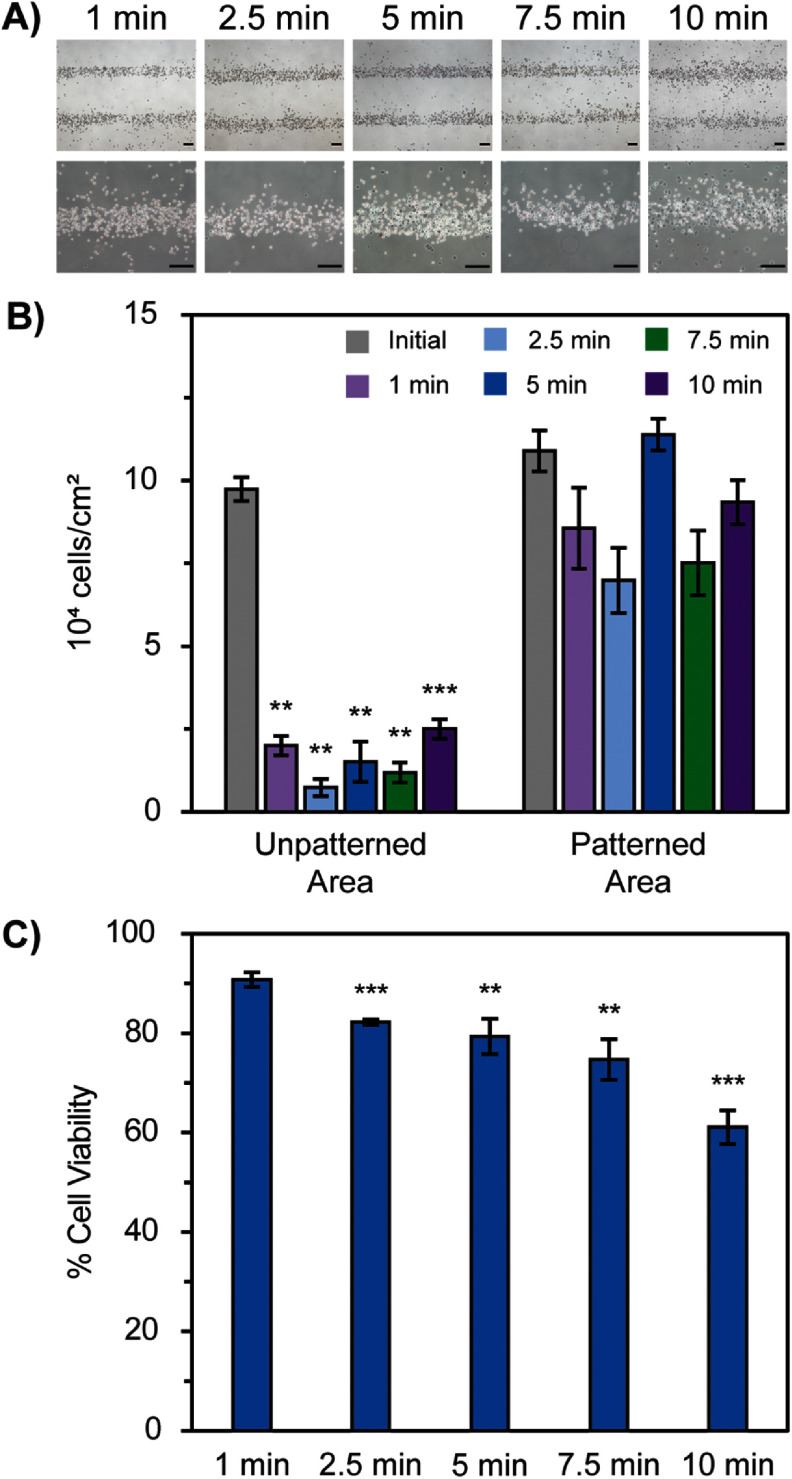
Centrifugation Time Affects Cell Adhesion and Viability of H9C2 Cells. (A) Representative images of H9C2 cells patterned in 200 μm line width patterns and washed with centrifugation at 43.3 RCF for 1, 2.5, 5, 7.5, and 10 min time periods using brightfield microscopy and 4x magnification (top row). Samples were stained with Trypan Blue to determine the percentage of cells that had damage to their membranes (bottom row). (B) Cell adherence within the patterned and unpatterned areas of each image before and after centrifugation washing of the conditions described in (A). Data is shown as mean ± standard deviation of n = 3 independent samples for each time period tested. Significance testing compared the initial cell adherence in each region before and after centrifugation at each time period using a paired two-group t-test (* p < 0.05, ** p < 0.01, *** p < 0.001). The absence of * is considered a nonsignificant p-value comparison. (C) Cell viability of patterns determined from Trypan Blue staining after centrifugation washing of the conditions described in (A). Data is shown as mean ± standard deviation of n = 3 independent samples for each time period tested. Significance testing compared the cell viability after centrifugation at 2.5 min, 5 min, 7.5 min, and 10 min to the lowest time period of 1 min using an independent two-group t-test (* p < 0.05, ** p < 0.01, *** p < 0.001). The absence of * is considered a nonsignificant p-value comparison. All scale bars represent 200 μm.

Similar results were seen when studying optimal centrifugation time while holding speed constant at 43.3 RCF. There was not a significant difference in the percentage of cells retained in the patterned areas after centrifugation for all time periods; however, all time periods tested showed a significant difference in the percentage of cells retained in the unpatterned areas (figures 4(A) and (B)).

All of the centrifugation speeds (43.3, 173, and 390 RCF) and times (1, 2.5, 5, 7.5, and 10 min) tested are all appropriate for the removal of cells in the unpatterned areas while retaining cells in the patterned areas. Therefore, determining the appropriate speed and time settings is largely dependent on the cell viability after centrifugation. Settings of 43.3 RCF for a 1 min time interval is determined to be the optimal washing system because the cell viability is significantly greater for these compared to all subsequent speeds and times tested. Trypan Blue staining increases with increasing centrifugation speed and time, indicating that there is a loss in cell membrane integrity. This result is likely due to the adhesion strength of the cell to the patterned area of the slide with biotin-streptavidin being greater than the force applied to the cell through centrifugation, so at high centrifugation speeds or time periods, the cell is stretched but cannot detach from the slide, causing its membrane to rupture.

**Figure 5.**
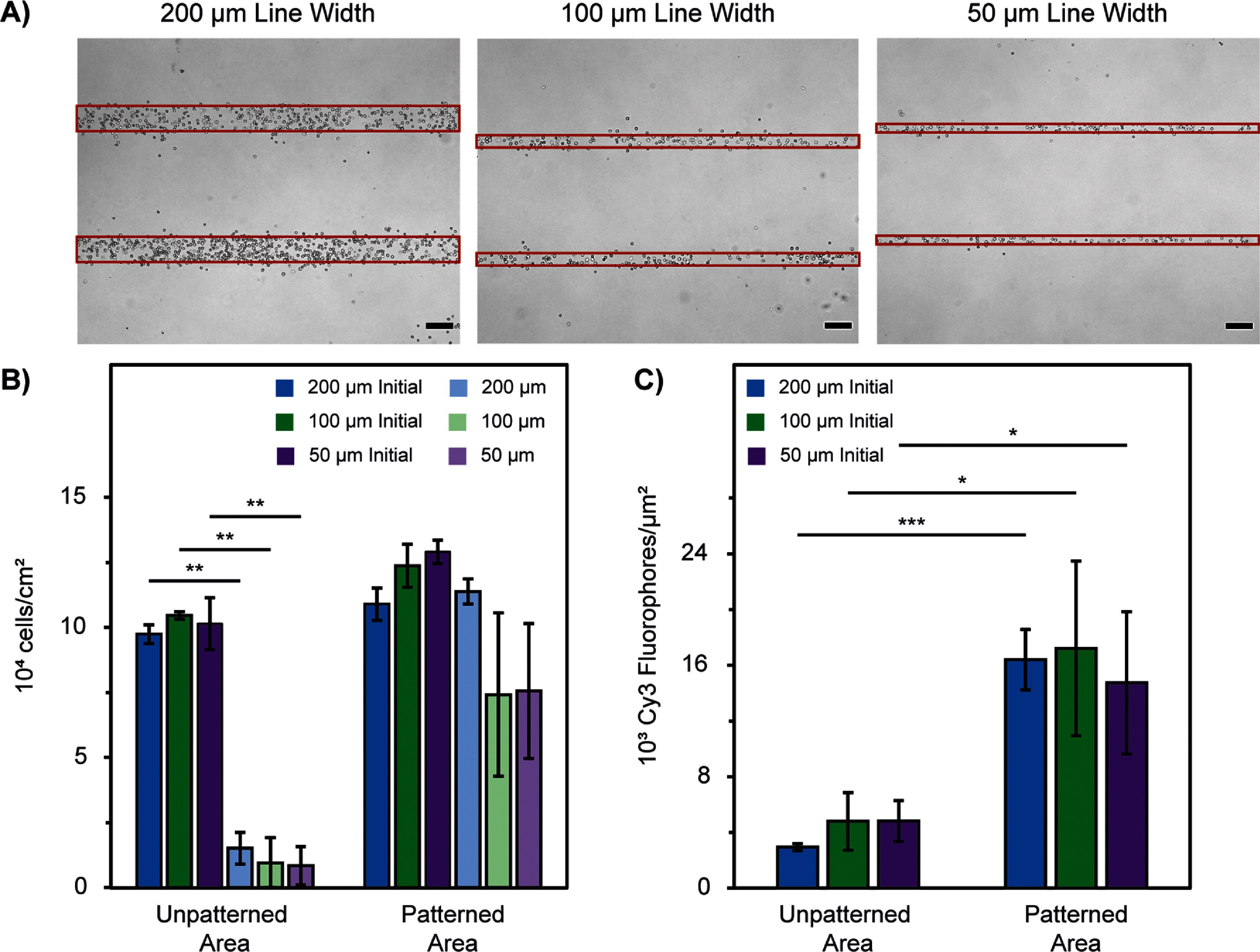
Centrifugation for Cell Patterns of Various Line Widths. (A) Representative images of H9C2 cells patterned in 50, 100, and 200 μm line width patterns and washed with centrifugation at 43.3 RCF in 1 min increments using brightfield microscopy and 4x magnification. The line width patterned areas of the image are denoted in red. (B) Cell adherence within the patterned and unpatterned areas of each image before and after centrifugation washing for each line width described in (A). Data is shown as mean ± standard deviation of n = 3 independent samples for each line width. Significance testing compared the cell adherence in each area before and after centrifugation for each line width using a paired two-group t-test (* p < 0.05, ** p < 0.01, *** p < 0.001). The absence of * is considered a nonsignificant p-value comparison. (C) Cy3 fluorophore concentration in the patterned and unpatterned areas of the sample for each line width described in (A). Data is shown as mean ± standard deviation for n = 4 patterned and unpatterned regions of each sample. Significance testing compared the Cy3 fluorophore concentration in the two regions of each line width described in (A) using an independent two-group t-test (* p < 0.05, ** p < 0.01, *** p < 0.001). The absence of * is considered a nonsignificant p-value comparison. All scale bars represent 200 μm.

As cell patterns are often required to achieve cell structures with long-term function, it is important to maintain high cell viability throughout the patterning process, where cell washing systems are often the most rigorous part of the process. Because there is often some variability in cell adhesion to any substrate, even patterned surfaces, one wash may not prove sufficient to achieve the desired pattern clarity. The same cell population can exhibit heterogenous behavior in relation to morphology and cell adhesion, especially when studied on different ECM substrate materials [73]. There are natural variations in receptor expression levels between individual cells that could be linked to adhesion strength [74] and cells in different cell culture stages can exhibit variations in their adhesive and mechanical properties [75, 76]. Additionally, there is variability in Cy3 fluorophore concentration in the patterned regions of each line width patterned (figure 5(C)), directly correlating to the biotin-streptavidin conjugated to the substrate; consequently, the strength of cell adhesion depends on the concentration of the biotin-streptavidin present on the surface. When an additional 1 min wash was tested (2 × 1 min) at 43.3 RCF, it was seen that both cell retention and cell viability in all regions was greater compared to an uninterrupted 2 min wash, although not significant (supplementary figures 8(B) and (C)). As a result, successive washes in 1 min increments are acceptable to improve this clarity without drastically changing cell retention in the patterned areas or cell viability (supplementary figure 8(C)).

### 3.4. Versatility of centrifugation in patterning multiple structures

Centrifugation is a successful washing approach to achieve clear cell patterns with three distinct line width sizes of 200, 100, and 50 μm. We demonstrate the versatility of this washing approach through both large-scale surface patterns (figure 1(B)) and through varying the sizes of these pattern structures (figure 5(A)).

The centrifugation washing system performs as desired for each pattern size, significantly removing cells in the unpatterned areas while retaining cells in the patterned areas (figure 5(B)). Overall, these results indicate that the patterning and centrifugation washing system is robust and can be used to achieve a variety of pattern sizes depending on the intended application.

Although not significantly different, there was an increase in variability in the 50 and 100 μm line width patterns compared to the 200 μm line width. Similar results were seen when analyzing the Cy3 fluorophore concentration, indicative of the streptavidin surface concentration on the slide. There were significant differences in fluorophore concentration when comparing the patterned and unpatterned areas of each line size, while the line size did not have a significant relationship with the concentration of the fluorophore in the patterned or the unpatterned region (figure 5(C)). As the line width decreases, a greater proportion of adhered cells reside on pattern edges with only a portion of their surface anchored with biotin-streptavidin, which likely weakens and creates variability in their adhesion and surface retention.

### 3.5. Cell fate and functionality is unaffected by patterning + centrifugation

Cell membrane integrity and esterase activity were used to evaluate the viability of a variety of cell types with our optimal patterning and centrifugation system. Since cells experience a force of adhesion from cell patterning as well as a force of centrifugation from patterning washing, there is opportunity for cell stretching and cell membrane rupture. Viability was verified using fully conjugated samples whose area represented a patterned surface washed with centrifugation (patterning + centrifugation) or a fully unconjugated surface (negative control). Calcein AM was used as an indicator of esterase activity and a live cell, while Eth-1 was used as an indicator of a disrupted cell membrane and a dead cell for H9C2s, MPCs, and HUVECs.

**Figure 6.**
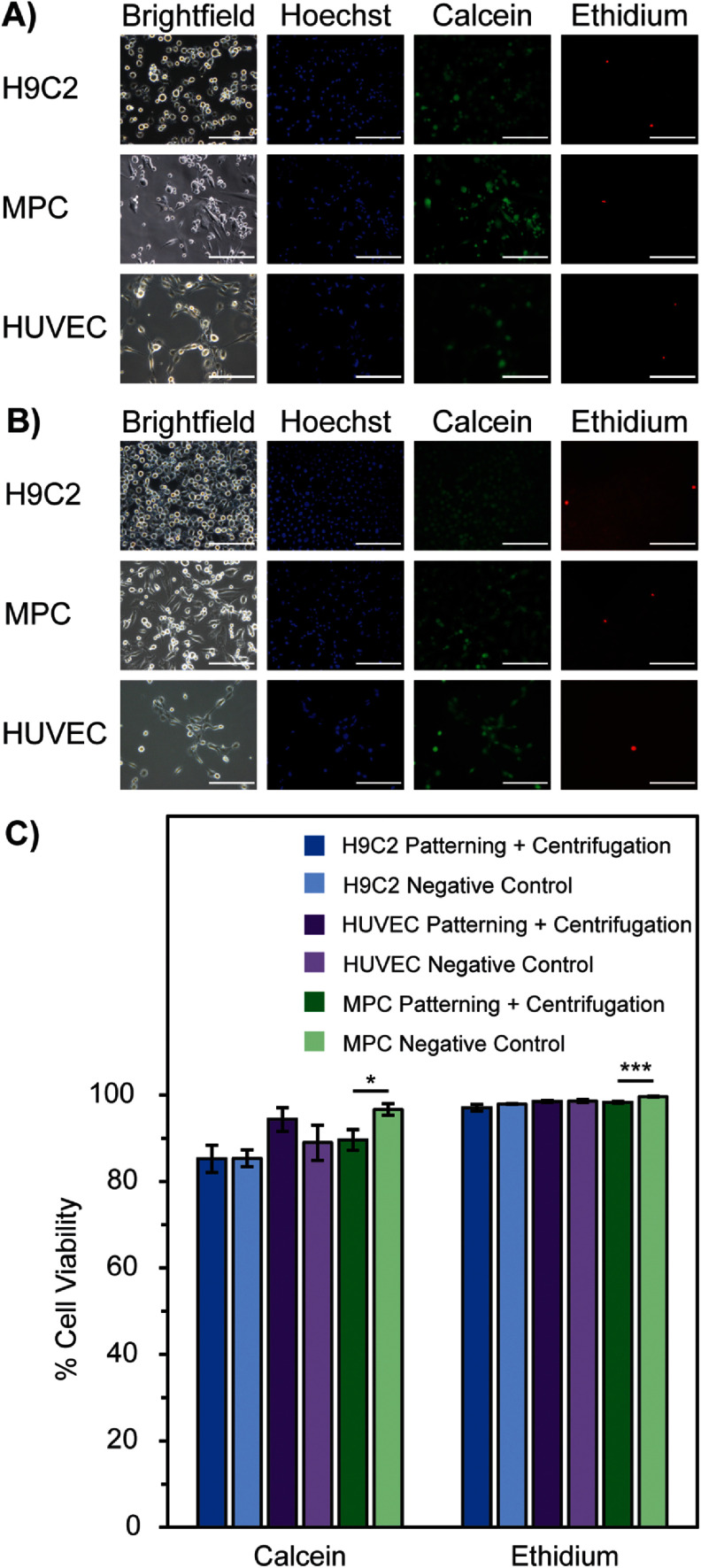
Short-Term Viability Following Patterning + Centrifugation. (A) Representative images of each cell type seeded directly onto their respective unconjugated culture surface (negative control) in cell media for 2 h then stained with Hoechst 33342, calcein AM, and ethidium homodimer-1 to determine cell viability. (B) Representative images of each cell type after seeding on their respective culture surface fully conjugated with biotin-streptavidin, then centrifugation at 2 × 1 min at 43.3 RCF (patterning + centrifugation control). The samples were then stained with Hoechst 33342, calcein AM, and ethidium homodimer-1 to determine cell viability. (C) Cell viability for each cell type under the conditions described in (A) and (B). Data is shown as mean ± standard deviation of n = 3 independent samples for each cell type and condition. Significance testing compared the cell viability of each condition in (A) and (B) for calcein and ethidium viability tests using an independent two-group t-test (* p < 0.05, ** p < 0.01, *** p < 0.001). The absence of * is considered a nonsignificant p-value comparison. All scale bars represent 200 μm.

All cell types maintain a high cell viability above 80% with calcein AM and above 90% Eth-1 when stained immediately after patterning + centrifugation washing at 43.3 RCF for 2 × 1 min, comparable to the negative control of unmodified cells seeded on their respective unconjugated surfaces (figure 6).

This observation of high cell viability indicates that the biotin-streptavidin patterning and centrifugation washing processes do not significantly alter the metabolic activity or the membrane integrity of the cells. As a result, there is potential to pattern a variety of cell types using adhesive patterning with biotin-streptavidin and using centrifugation washing without immediately affecting cell health and function. While there is a significant difference in the number of viable MPCs between the tested conditions for both stains, which is not seen with H9C2 or HUVECs, it is likely due to MPCs being a human primary cell line which is more sensitive to environmental changes compared to the two animal cell lines. The addition of 1% glucose and 1% penicillin-streptomycin in the PBS solutions in the patterning process is used to try to combat this challenge. However, the overall viability of MPCs in the patterning + centrifugation control is still above 80% with the calcein AM stain and above 90% with the Eth-1 stain, indicating no overall concern for cell health.

**Figure 7.**
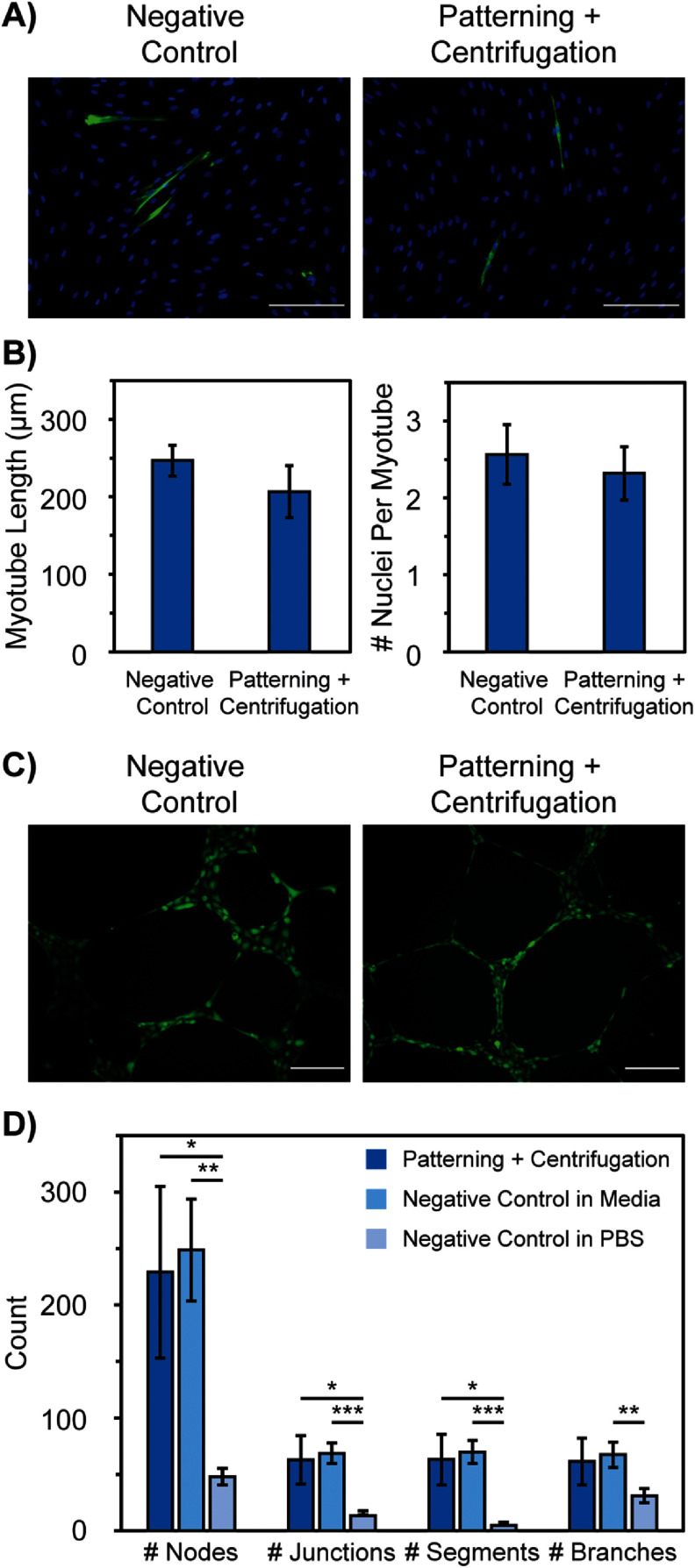
Long-Term Functionality through Tube Formation Assays. (A) Representative images of MPCs seeded on collagen 1 in differentiation media directly from culture (negative control, left) and of MPCs seeded on collagen 1 fully conjugated with biotin-streptavidin and centrifuged 2 × 1 min at 43.3 RCF then placed in differentiation media (patterning + centrifugation, right). Samples were fixed on Day 8 and stained with anti-myosin (green) and DAPI (blue), then imaged using fluorescent microscopy at 10x magnification to visualize tube formation. (B) Average myotube length and number of nuclei per myotube for each condition described in (A). Data is shown as mean ± standard deviation of n = 3 independent samples for each condition. Significance testing compared the negative control to patterning + centrifugation using a two-group independent t-test (* p < 0.05, ** p < 0.01, *** p < 0.001). The absence of * is considered a nonsignificant p-value comparison. (C) Representative images of HUVEC cells seeded on Matrigel in EGM^TM −2^ BulletKit^TM^ media directly from culture (negative control, left) and of HUVECs seeded on Matrigel fully conjugated with biotin-streptavidin and centrifuged 2 × 1 min at 43.3 RCF then placed in EGM^TM −2^ media (patterning + centrifugation, right). Samples were left in media overnight then stained with Calcein AM and imaged using fluorescent microscopy at 10x magnification to visualize tube formation. (D) Quantitative analysis of HUVEC tube formation including the number of nodes, junctions, segments, and branches formed from samples of each condition in (C). Data is shown as mean ± standard deviation of n = 3 independent samples for each condition. Significance testing compared the negative control in media, the negative control in PBS, and the patterning + centrifugation for each metric using a two-group independent t-test (* p < 0.05, ** p < 0.01, *** p < 0.001). The absence of * is considered a nonsignificant p-value comparison. All scale bars represent 200 μm.

MPCs and HUVECs were chosen for longer term function studies, as they are cell lines that naturally form tube structures and can give vital information on the long-term impacts of the patterning and centrifugation processes. Both MPCs and HUVECs form tube structures after patterning and centrifugation, further verifying that cell health is not greatly impacted and cells are not confined from migrating and behaving naturally (figure 7).

There was no significant difference in the myotube length or the number of nuclei per myotube when comparing MPCs placed in differentiation media after patterning + centrifugation washing compared to the negative control (figure 7(B)). Similarly, there was no significant differences in the HUVEC tube formation metrics studied (number of nodes, junctions, segments, or branches) when placed in EGM^TM −2^ BulletKit^TM^ media after patterning + centrifugation washing compared to the negative control (figure 7(D)). The tube formation metrics studied for MPCs [77–79] as well as HUVECs [80–83] are comparable to others found in literature, indicating that the addition of biotin to the cell surface and the biotin-streptavidin conjugations on the slide surface do not alter the way the cells naturally behave or interact with one another. There is not a significant difference in the number of branches between HUVECs that are patterned and centrifuged compared to a control of HUVECs seeded on unconjugated Matrigel in PBS. It is not surprising that some branch formation is seen in PBS because branch formation is the first step in HUVEC tube formation. Branches are usually formed within 1–2 h after HUVECs are incubated with media on Matrigel, but they often do not form complete tubes until at least 4 h. Therefore, it is possible that the cells begin to form these branch structures to start tube formation in PBS but are unable to form complete tube structures without the addition of the EGM^TM −2^ BulletKit^TM^ media. Overall, these results motivate the use of our patterning and centrifugation system for initial cell placement of any cell type on an appropriate substrate. Cell patterning using this technique only temporarily adheres cells to the pattern location, but does not immobilize them long-term to prevent their biological function.

## 4. Conclusions

The application of centrifugation in adhesive biological systems to create organized cell structures is a previously untapped area explored through this work. The main drawback to current adhesive cell patterning systems is the unreliability of washing approaches to remove cells weakly adhered in the unpatterned regions while maintaining cells strongly adhered in the patterned regions. This study shows that centrifugation is a robust method that can be used to wash away weakly adhered cells to create distinct cell patterns using adhesive patterning with biotin-streptavidin. Both centrifugation and shear flow washing methods remove significantly more cells in the unpatterned regions compared to the patterned regions; however, centrifugation also retains 33.6% and 26.1% more of the initially-bound cells within the desired patterns compared to shear flow parallel and perpendicular to the patterned structures, respectively. This improvement of cell pattern density may be necessary in some applications to achieve a desired long-term biological result. The force needed to detach the cells from the slide in the unpatterned regions is fundamentally intuitive, based on the properties of the cell and media, as well as the relative centrifugal force, a key advantage over other more complex washing approaches using microfluidic shear flow devices. The centrifugation system is easy to set up and operate, has precise temperature control for variable cellular environmental needs, and is reproducible with attachments already available for common benchtop centrifuges. This study demonstrates that adhesive patterning using biotin-streptavidin in combination with centrifugation washing produces cell patterns in a variety of sizes, with potential to tailor to additional geometries to fit a specific application. Cells that are patterned and centrifuged have shown little changes to viability or long-term function, as shown in studies of tube-forming cell types. Therefore, centrifugation can be applied to a variety of adhesive biological systems, regardless of the binding motif, to create organized cell structures that still deliver a necessary functional outcome.

## Data Availability

The data that support the findings of this study are openly available at the following URL/DOI: https://doi.org/10.18126/g7qt-bp50.
